# Supplementary material for: Phase analysis on the error scaling of entangled qubits in a 53-qubit system
Source: Sci Rep. 2021 Jul 14;11:14491. doi: 10.1038/s41598-021-93856-8 (PMC8280220; doi:10.1038/s41598-021-93856-8)
Supplement: Supplementary file 1 — Supplementary Information. [file 41598_2021_93856_MOESM1_ESM.docx]

**Phase Analysis on the Error Scaling of Entangled Qubits in a 53-Qubit System**

Wei-Jia Huang^1^, Wei-Chen Chien^2^, Chien-Hung Cho^1^, Che-Chun Huang^1^, Tsung-Wei Huang ^3^, Seng Ghee Tan^4^, C. Cao^5^, Bei Zeng^5^, and Ching-Ray Chang^2,6^

^1^*Department of Physics, National Taiwan University and Hon Hai Research Institute, Taipei, Taiwan*

*^2^Graduate Institute of Applied Physics, National Taiwan University, Taipei, Taiwan*

^3^*Department of information and computer Engineering, Chung Yuan Christian University, Taiwan*

^4^*Department of Optoelectric Physics, Chinese Culture University, 55 Hwa-Kang Road, Yang-Ming-Shan, Taipei 11114, Taiwan*

*^5^Department of Physics, The Hong Kong University of Science and Technology, Clear Water Bay, Kowloon, Hong Kong, China*

*^6^Graduate Institute of Applied Physics and NTU-IBM Quantum Hub, National Taiwan University, Taipei, Taiwan*

Ching-Ray Chang

Email: [crchang@phys.ntu.edu.tw](mailto:crchang@phys.ntu.edu.tw)

Bei Zeng

Email: [zengb@ust.hk](mailto:zengb@ust.hk)

This file includes

Supplement A,B,C,D

Figures SA, SB, SC1, SC2, SC3, SC4,SD.

Correspondence to: [crchang@phys.ntu.edu.tw](mailto:crchang@phys.ntu.edu.tw)

**Supplementary Information**

**Supplement A**


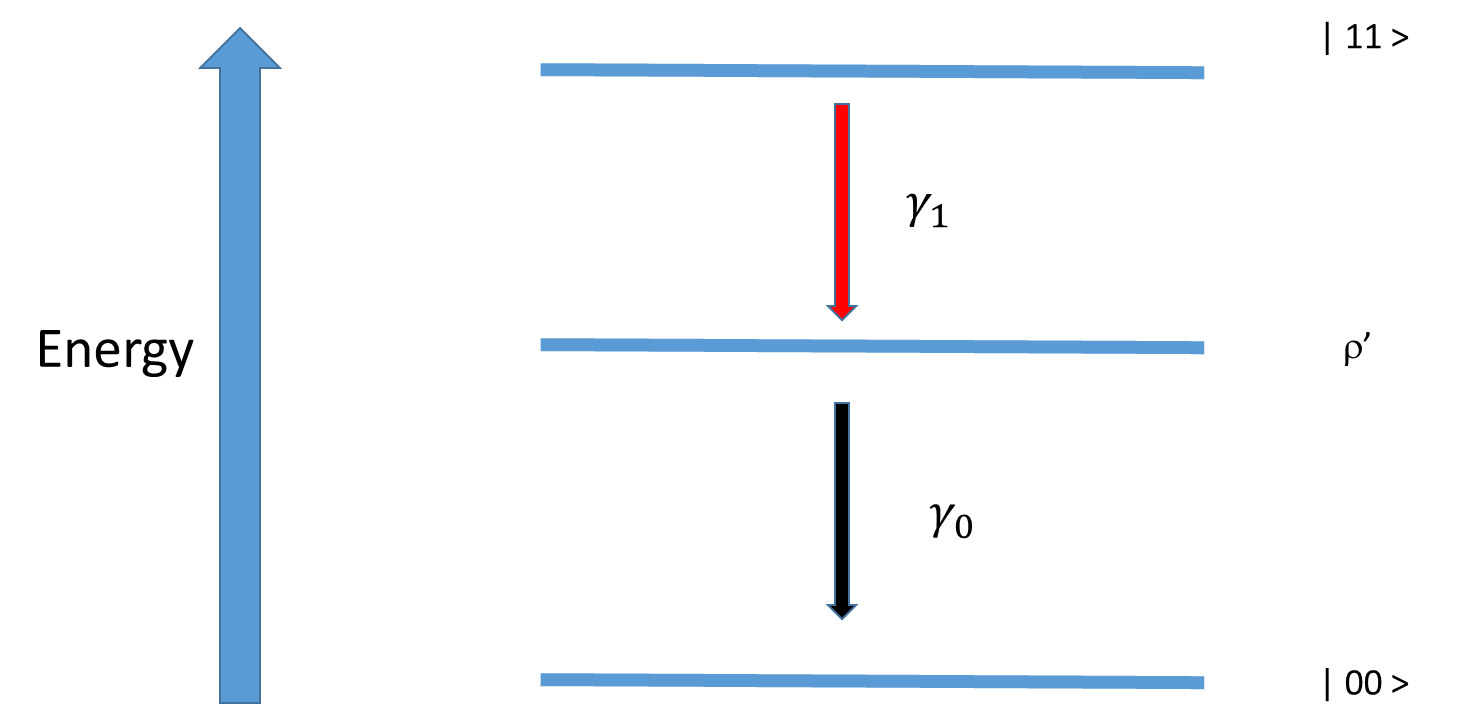


Figure SA: Four states |11>, |01>, |10> and |00> of a 2-qubit pair. $\rho'$ is a quantum state in the subspace spanned by $\left| 01\rangle\right.$and $\left| 10\rangle\right.$, which is usually mixed. It can be written as

$\rho'=\left( \begin{matrix} 0 & 0 & 0 & 0 \\ 0 & a & re^{-i\theta} & 0 \\ 0 & re^{i\theta} & 1-a & 0 \\ 0 & 0 & 0 & 0 \end{matrix} \right)$,

Here *r*, *a*, *θ* are parameters that determines the density matrix of *ρ'*. Possible transitions between states are shown for an energy dissipative system, and the ground state |00> is assumed to be always alive. γ_1_ represents the transition rate from |11> to $\rho'$, while γ_0_ is from $\rho'$ to |00>.

**Supplement B**


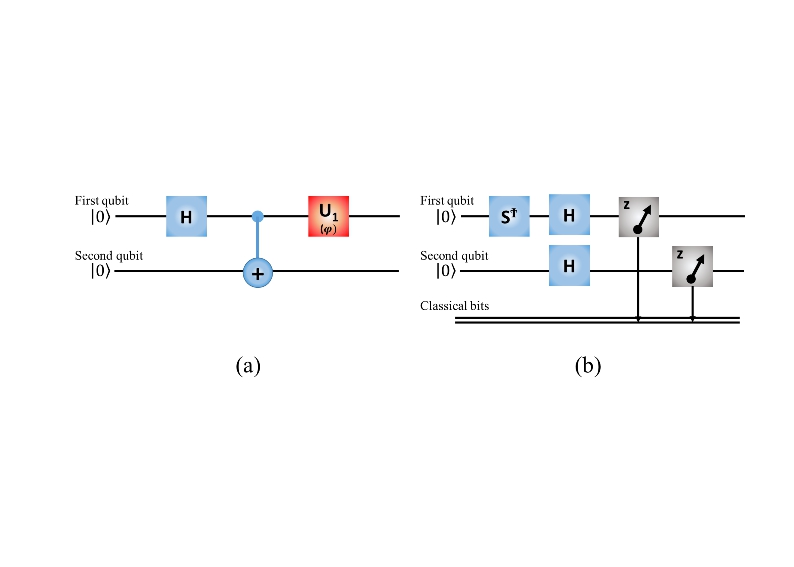


Figure SB: Operating on q[0]⊗q[1](first qubit and second qubit) with the Hadamard gate and the CNOT gate.

After operating on $q\left[ 0 \right]\otimes q\left[ 1 \right]$ with the Hadamard gate and the CNOT gate, possible transitions in a noisy environment are shown in Fig. SB. The resultant state will usually be not $\frac{1}{\sqrt{2}}(\left| 00\rangle\right.+\left| 11\rangle\right.)$, the state first assigned to the quantum computer. The noise-induced transitions generate $q\left[ 0 \right]\otimes q\left[ 1 \right]= A\left| 00>+B \right|01>+C|10>+D|11>$. However, operator $U1(\varphi)=\left( \begin{matrix} 1 & 0 \\ 0 & e^{i\varphi} \end{matrix} \right)$ acting on this state will impart a phase of $e^{i\varphi}$ to state |1> in q[0]. This usually results in the final state of $A\left| 00>+B \right|01>+Ce^{i\varphi}|10>+De^{i\varphi}|11>$.

**Supplement C**

In this section, we present a method with uncorrelated errors. We know if the circuit shown in Fig. 1 is noiseless, the corresponding circle in Fig. 2 would be the largest circle with radius $\sqrt{{|\left\langle\Psi\left| W_{2} \right|\Psi\right\rangle|}^{2}+{|\left\langle\Psi\left| W_{2}^{'} \right|\Psi\right\rangle|}^{2}}=2\sqrt{2}$. The circuit is accompanied by uncorrelated noise, as shown in Fig. SC1, where we have considered models for three different noise sources: the depolarizing channel, the dephasing channel and the amplitude damping channel^32,33^.


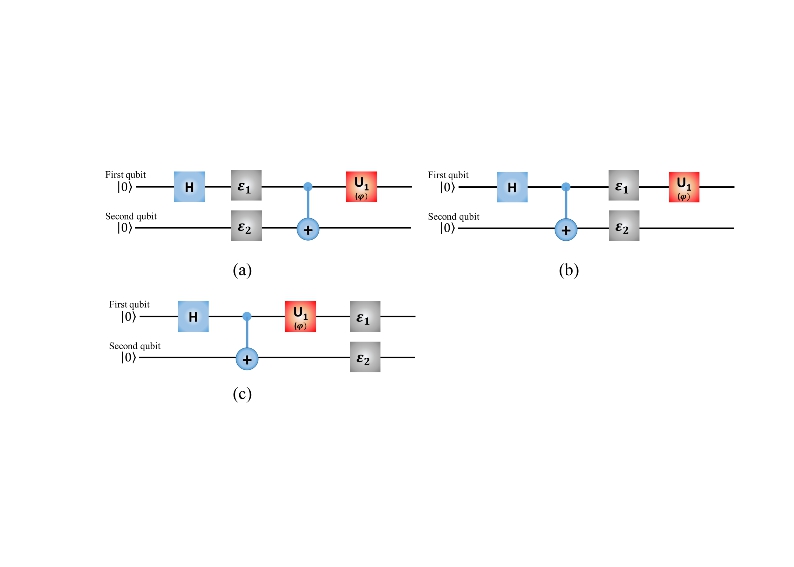


Figure SC1: Noisy Circuit with noise channels $\varepsilon_{1}$ and $\varepsilon_{2}$. (a) Noise before the CNOT gate. (b) Noise after the CNOT gate. (c) Noise after the phase rotation gate.

Denote the noise rate as *p*, the depolarizing noise channel is modeled by

$\rho\to\left( 1-p \right)\rho+ pI/2$

The dephasing noise is modeled by

$\rho\to\sum_{i} E_{i}\rho E_{i}$

where $E_{0}=\left( \begin{matrix} \sqrt{1-p} & 0 \\ 0 & \sqrt{1-p} \end{matrix} \right)$, $E_{1}=\left( \begin{matrix} \sqrt{p} & 0 \\ 0 & 0 \end{matrix} \right)$,$E_{2}=\left( \begin{matrix} 0 & 0 \\ 0 & \sqrt{p} \end{matrix} \right)$. The amplitude damping noise is modeled by

$\rho\to A_{0}\rho A_{0}+ A_{1}\rho A_{1}$

where $A_{0}=\left( \begin{matrix} 1 & 0 \\ 0 & \sqrt{1-p} \end{matrix} \right)$,$A_{1}=\left( \begin{matrix} 0 & \sqrt{p} \\ 0 & 0 \end{matrix} \right)$. Suppose $\rho$ is the density matrix of the state generated from the noisy circuit, $p_{1}$ and $p_{2}$ are the noise rates of channels $\varepsilon_{1}$ and $\varepsilon_{2}$ , respectively.

When the noise channel is located prior to the CNOT gate,

the density matrix for the depolarizing noise is given by

$\rho=\left( \begin{matrix} \frac{1}{2}-\frac{1}{4}p_{2} & 0 & 0 & (\frac{1}{2}-\frac{1}{2}p_{1}-\frac{1}{4}p_{2}+\frac{1}{4}p_{1}p_{2})e^{-i\varphi} \\ 0 & \frac{1}{4}p_{2} & \left( \frac{1}{4}p_{2}-\frac{1}{4}p_{1}p_{2} \right)e^{-i\varphi} & 0 \\ 0 & (\frac{1}{4}p_{2}-\frac{1}{4}p_{1}p_{2})e^{i\varphi} & \frac{1}{4}p_{2} & 0 \\ (\frac{1}{2}-\frac{1}{2}p_{1}-\frac{1}{4}p_{2}+\frac{1}{4}p_{1}p_{2})e^{i\varphi} & 0 & 0 & \frac{1}{2}-\frac{1}{4}p_{2} \end{matrix} \right)$

Notice that here $the excited state \rho^{'}=\left( \begin{matrix} 0 & 0 & 0 & 0 \\ 0 & a & re^{-i\theta} & 0 \\ 0 & re^{i\theta} & 1-a & 0 \\ 0 & 0 & 0 & 0 \end{matrix} \right)$ has parameters $a=\frac{1}{2}, r=\frac{1}{2}-\frac{1}{2}p_{1},\theta=\varphi$,

which means $\rho'=\left( \begin{matrix} 0 & 0 & 0 & 0 \\ 0 & \frac{1}{2} & \left( \frac{1}{2}-\frac{1}{2}p_{1} \right)e^{-i\varphi} & 0 \\ 0 & \left( \frac{1}{2}-\frac{1}{2}p_{1} \right)e^{i\varphi} & \frac{1}{2} & 0 \\ 0 & 0 & 0 & 0 \end{matrix} \right)$.

A special case is if $p_{1}$ = 0, then $\rho^{'}=|\left. \Psi^{'} \right\rangle\left\langle\Psi^{'} \right.|$ with $\left| \Psi'\rangle\right.=\frac{1}{\sqrt{2}}(\left| 01\rangle\right.+e^{i\varphi}\left| 10\rangle\right.)$.

The density matrix for the dephasing noise is

$$\rho=\left( \begin{matrix} \frac{1}{2} & 0 & 0 & (\frac{1}{2}-\frac{1}{2}p_{1})e^{-i\varphi} \\ 0 & 0 & 0 & 0 \\ 0 & 0 & 0 & 0 \\ (\frac{1}{2}-\frac{1}{2}p_{1})e^{i\varphi} & 0 & 0 & \frac{1}{2} \end{matrix} \right)$$

Last, the density matrix for the amplitude damping noise is

$$\rho=\left( \begin{matrix} \frac{1}{2}+\frac{1}{2}p_{1} & 0 & 0 & \frac{1}{2}\sqrt{1-p_{1}}e^{-i\varphi} \\ 0 & 0 & 0 & 0 \\ 0 & 0 & 0 & 0 \\ \frac{1}{2}\sqrt{1-p_{1}}e^{i\varphi} & 0 & 0 & \frac{1}{2}-\frac{1}{2}p_{1} \end{matrix} \right)$$

When the noise channel is located after the CNOT gate or the phase rotation gate, the density matrix for the depolarizing noise is given by

$\rho=\left( \begin{matrix} \frac{1}{2}-\frac{1}{4}p_{1}-\frac{1}{4}p_{2}+\frac{1}{4}p_{1}p_{2} & 0 & 0 & (\frac{1}{2}-\frac{1}{2}p_{1}-\frac{1}{2}p_{2}+\frac{1}{2}p_{1}p_{2})e^{-i\varphi} \\ 0 & \frac{1}{4}p_{1}+\frac{1}{4}p_{2}-\frac{1}{4}p_{1}p_{2} & 0 & 0 \\ 0 & 0 & \frac{1}{4}p_{1}+\frac{1}{4}p_{2}-\frac{1}{4}p_{1}p_{2} & 0 \\ (\frac{1}{2}-\frac{1}{2}p_{1}-\frac{1}{2}p_{2}+\frac{1}{2}p_{1}p_{2})e^{i\varphi} & 0 & 0 & \frac{1}{2}-\frac{1}{4}p_{1}-\frac{1}{4}p_{2}+\frac{1}{4}p_{1}p_{2} \end{matrix} \right)$

Notice that here for the excited state $\rho'$, $a=\frac{1}{2}, r=0. \rho'=\left( \begin{matrix} 0 & 0 & 0 & 0 \\ 0 & \frac{1}{2} & 0 & 0 \\ 0 & 0 & \frac{1}{2} & 0 \\ 0 & 0 & 0 & 0 \end{matrix} \right)$.

The density matrix for the dephasing noise is given by

$$\rho=\left( \begin{matrix} \frac{1}{2} & 0 & 0 & (\frac{1}{2}-\frac{1}{2}p_{1}-\frac{1}{2}p_{2}+\frac{1}{2}p_{1}p_{2})e^{-i\varphi} \\ 0 & 0 & 0 & 0 \\ 0 & 0 & 0 & 0 \\ (\frac{1}{2}-\frac{1}{2}p_{1}-\frac{1}{2}p_{2}+\frac{1}{2}p_{1}p_{2})e^{i\varphi} & 0 & 0 & \frac{1}{2} \end{matrix} \right)$$

Last, the density matrix for the amplitude damping noise is

$$\rho=\left( \begin{matrix} \frac{1}{2}+\frac{1}{2}p_{1}p_{2} & 0 & 0 & \frac{1}{2}\sqrt{1-p_{1}-p_{2}+p_{1}p_{2}}e^{-i\varphi} \\ 0 & \frac{1}{2}p_{1}-\frac{1}{2}p_{1}p_{2} & 0 & 0 \\ 0 & 0 & \frac{1}{2}p_{2}-\frac{1}{2}p_{1}p_{2} & 0 \\ \frac{1}{2}\sqrt{1-p_{1}-p_{2}+p_{1}p_{2}}e^{i\varphi} & 0 & 0 & \frac{1}{2}-\frac{1}{2}p_{1}-\frac{1}{2}p_{2}+\frac{1}{2}p_{1}p_{2} \end{matrix} \right)$$

Notice that here the parameters for $\rho^{'}$are $a=\frac{\frac{1}{2}p_{1}-\frac{1}{2}p_{1}p_{2}}{\frac{1}{2}p_{1}+\frac{1}{2}p_{2}-p_{1}p_{2}}, r=0.$

We conclude that if there is depolarizing noise before CNOT gate (which is usually true in experiments), $\theta=\varphi$.

Therefore, we can easily calculate the relation between radius $R= \sqrt{{tr}^{2}(\rho W_{2})+{tr}^{2}(\rho W_{2}^{'})}$ and noise rates $p_{1}$, $p_{2}$. For example, when the amplitude damping channel is located after the CNOT gate, $R=2\sqrt{2(1-p_{1}-p_{2}+p_{1}p_{2})}$. In addition to the depolarizing noise, in the case of noise before the CNOT gate, R does not depend on the phase ϕ, therefore the phase trajectory is always a circle with radius R for different noise channels and noise rates. If we assume $p=p_{1}=p_{2}$, the radii for the different noise channels and noise rates will be as shown in Fig. SC2.

1. (b)

Fig SC2: Radii for different noise channels and noise rates. (a) Result for system of noise channel located before the CNOT gate. (b) Result for system of noise channel located after the CNOT gate or after the phase rotation gate.

If the amplitude damping channel is located after the CNOT or the phase rotation gate, we will have $p=1- \frac{R}{2\sqrt{2}}$. It is also known that noise rate is $p=1- e^{-\frac{t}{T_{1}}}$ for the amplitude damping noise. From the two equations above, we have $\ln\left( \frac{2\sqrt{2}}{R} \right)=\frac{t}{T_{1}}$, which is consistent with the simulation results in Fig.2(b). The trajectories of the noise rates in Fig. SC3 correspond to the trajectories in Fig. 2(b) with different $T_{1}$.

Fig SC3: Phase trajectories for the uncorrelated amplitude damping channels located after the CNOT/phase gate.

When the depolarizing channel is located before the CNOT gate, the trajectory will be ellipses instead of circles, as shown in Fig SC4.

Fig SC4: Phase trajectories for the uncorrelated depolarizing channel is located before CNOT gate.

We will now also take $T_{2}$ into consideration. If the noise channel is a combination of the amplitude damping noise and the dephasing noise, it will transform a single qubit density matrix through

$\rho= \left( \begin{matrix} {1-\rho}_{11} & \rho_{01} \\ {\rho_{01}}^{*} & \rho_{11} \end{matrix} \right)\to\left( \begin{matrix} 1-\rho_{11}e^{-\frac{t}{T_{1}}} & \rho_{01}e^{-\frac{t}{T_{2}}} \\ {\rho_{01}}^{*}e^{-\frac{t}{T_{2}}} & \rho_{11}e^{-\frac{t}{T_{1}}} \end{matrix} \right)$.

For a 2-qubit state $\left| \Psi\rangle\langle\Psi\right.|$, where $\left| \Psi\rangle\right.= \frac{1}{\sqrt{2}}(\left| 00\rangle\right.+e^{i\varphi}\left| 11\rangle\right.)$, we denote $T_{1}^{1}$ and $T_{2}^{1}$ as the relaxation time $T_{1}$ and dephasing time $T_{2}$ of the first qubit, $T_{1}^{2}$ and $T_{2}^{2}$ as, respectively, the “times” for the second qubit. The combined noise channel transforms $\left| \Psi\rangle\langle\Psi\right.|$ to

$$\rho=\left( \begin{matrix} 1-\frac{1}{2}e^{-\frac{t}{T_{1}^{1}}}-\frac{1}{2}e^{-\frac{t}{T_{1}^{2}}}+\frac{1}{2}e^{-\frac{t}{T_{1}^{1}}-\frac{t}{T_{1}^{2}}} & 0 & 0 & \frac{1}{2}e^{-\frac{t}{T_{2}^{1}}-\frac{t}{T_{2}^{2}}}e^{-i\varphi} \\ 0 & \frac{1}{2}e^{-\frac{t}{T_{1}^{2}}}-\frac{1}{2}e^{-\frac{t}{T_{1}^{1}}-\frac{t}{T_{1}^{2}}} & 0 & 0 \\ 0 & 0 & \frac{1}{2}e^{-\frac{t}{T_{1}^{1}}}-\frac{1}{2}e^{-\frac{t}{T_{1}^{1}}-\frac{t}{T_{1}^{2}}} & 0 \\ \frac{1}{2}e^{-\frac{t}{T_{2}^{1}}-\frac{t}{T_{2}^{2}}}e^{i\varphi} & 0 & 0 & \frac{1}{2}e^{-\frac{t}{T_{1}^{1}}-\frac{t}{T_{1}^{2}}} \end{matrix} \right)$$

The phase trajectories will still be circles.

**Supplement D**

**Classical simulation results of phase angle and orthogonal measurements for state** $\left| \Psi'\rangle\right.$


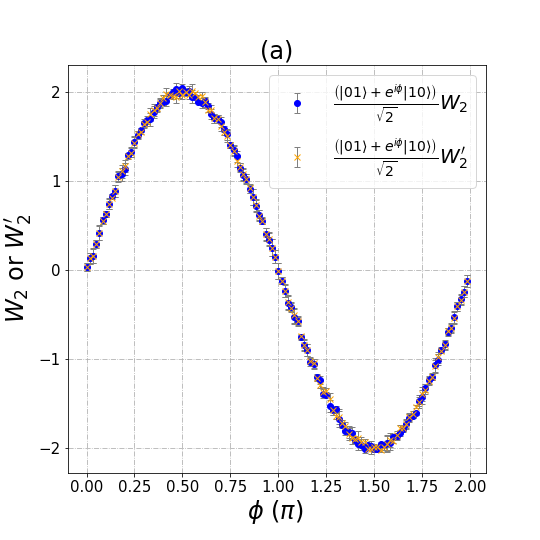

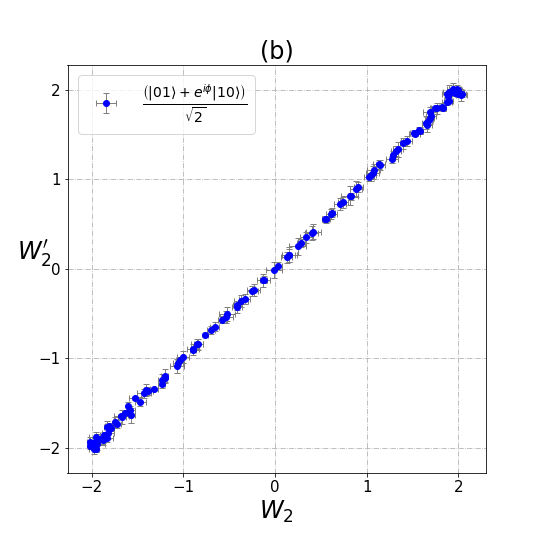
 Fig. SD: The classical simulation results of superposition states with $\left| \Psi'\rangle\right.=\frac{1}{\sqrt{2}}(\left| 01\rangle\right.+e^{i\varphi}\left| 10\rangle\right.)$. (a) The relationship of $\left\langle W_{2} \right\rangle$,$\left\langle W_{2}^{'} \right\rangle$ with phase angle *ϕ* . (b) The relationship between $\left\langle W_{2} \right\rangle$ a$\mathrm{nd}\left\langle W_{2}^{'} \right\rangle$.
